# Profiles of *Streptococcus thermophilus* MN‐ZLW‐002 nutrient requirements in controlled pH batch fermentations

**DOI:** 10.1002/mbo3.633

**Published:** 2018-04-22

**Authors:** Gefei Liu, Yali Qiao, Yanjiao Zhang, Cong Leng, Jiahui Sun, Hongyu Chen, Yan Zhang, Aili Li, Zhen Feng

**Affiliations:** ^1^ Key Laboratory of Dairy Science of Ministry of Education College of Food Science Northeast Agricultural University Harbin Heilongjiang China

**Keywords:** amino acid, inosine, purine, pyrimidine, requirement profiles, *S. thermophilus*, vitamin

## Abstract

This study aimed to evaluate the profiles of *Streptococcus thermophilus* nutrient requirements to guide the design of media for high cell density culturing. The growth kinetics, physiological state, and nutrient requirement profiles of *S. thermophilus* were analyzed in chemically defined media. The results showed that the intracellular ATP concentration, H^+^‐ATPase activity, NADH/NAD ^+^, and NH_3_ concentrations varied with intracellular pH. The nutrient components with the highest amounts required were Leu and Asp; ascorbic acid and *p*‐amino benzoic acid; K^+^ and PO_4_^3−^; and guanine and uracil. The nutrient components with the largest required ratios were Arg, His, and Met; folic acid, cyanocobalamine, biotin, and nicotinic acid; Ca^2+^ and Mg^2+^; and guanine and uracil. In this study, different nutrient components were primarily used at different phase. Trp, Tyr, calcium pantothenate, thiamine, guanine, and Mg^2+^ were mainly used from late‐lag to midexponential phase. Met, Pro, Phe, Ala, Gly, nicotinic acid, and riboflavin were mainly used from midexponential to late‐exponential phase. The highest bioavailabilities of nutrient components were also found at diverse phase. Met, Leu, Ile, Asn, Glu, Lys, Pro, Gly, riboflavin, nicotinic acid, adenine, uracil, inosine, and Ca^2+^ had the highest bioavailability from late‐lag to midexponential phase. Lactose, Glu, Asp, His, Trp, Cys, Val, Arg, Phe, Ala, Ser, Thr, Tyr, folate and cobalamin, calcium pantothenate, ascorbic acid, thiamine, biotin, *p*‐amino benzoic acid, vitamin B_6_, K^+^, Mg^2+^, guanine, xanthine, and PO_4_^3−^ had the highest bioavailability from midexponential to late‐exponential phase. This study elucidated the nutrient requirement profiles with culture time and biomass at various average growth rates during the growth of *S. thermophilus*. The present results will help to formulate complex media for high cell density cultivation and provide the theoretical basis for *S. thermophilus* feeding strategies.

## 1 INTRODUCTION

*Streptococcus thermophilus* is commonly used in the production of yogurt and cheese and is the second most important industrial dairy starter (Prajapati, Nathani, Patel, Senan, & Joshi, 2013). Regarding the preparation of inoculums, concentrated starter cultures have been increasingly used to directly inoculate food matrices. Thus, Direct‐Vat‐Set (DVS) has been widely used in the manufacture of fermented dairy products (Hansen, 2002). Among the steps of DVS preparation, high cell density culturing pivotally influences the quality of the starter (Buckenhüskes, 1993). *S. thermophilus* is a fastidious organism that requires carbohydrates, amino acids, vitamins, nucleotides, and minerals for growth in a defined medium. The complex nutrient requirements of this organism are typically met in growth media by the addition of undefined components, such as protein hydrolysates and yeast extract (Hongfei et al., 2013). However, current assays to assess nutrient requirements seldom consider those of *S. thermophilus*, limiting the usefulness of these tests to some extent.

Medium composition plays a crucial role in the high cell density culture of lactic acid bacteria (LAB). The effect of specific nutrients on LAB proliferation is dependent on the nutrient requirements of a given species (Hayek & Ibrahim, 2013). Thus, the design of media to obtain high cell density cultures of *S. thermophilus* requires a thorough understanding of the nutrient requirements of this bacterium. Many studies have focused on employing single omission and addition techniques to optimize the concentrations of essential and stimulatory compounds for LAB growth (Chengjie, Guojun, Zhenmin, Guangyu, & Zhengjun, 2016; Guiying, Mills, & Block, 2009; Letort & Juillard, 2001). Current knowledge of the nutrient requirements of *S. thermophilus* is insufficient, especially the consumption rate of amino acids, vitamins, ions, purines, and pyrimidine at various average growth rates with the culture time and biomass. This lack of reliable information has hindered the rational design of cultivation media and the optimization of bioprocesses (Lahtvee et al., 2011). Furthermore, the systematic and global study of the amino acids, vitamins, ions, purines, pyrimidine, inosine, and lactose requirements during *S. thermophilus* growth has never been reported.

Previous studies have shown that *S. thermophilus* MN‐ZLW‐002 (ST‐MZ‐2) displays excellent properties in cheese and yogurt starter. The goal of this study was to evaluate the amino acids, vitamins, ions, purines, pyrimidine, inosine, and lactose requirements for the growth of ST‐MZ‐2 by assessing average growth rates with respect to culture time and biomass yields to help design of a high cell density cultivation medium for ST‐MZ‐2 and study the metabolic mechanism of nutrients.

## 2 MATERIALS AND METHODS

### 2.1 Strain and growth conditions

ST‐MZ‐2 was obtained from a previous study (Xiaohong, Nan, Guoqing, Qi, & Lanwei, 2012). The cells were stored at −80°C in 12% sterile reconstituted skim milk containing 15% glycerol. Eight subcultivations were performed in chemically defined medium (CDM) for 12 hr at 42.5°C to obtain a stable growth response. The CDM (the initial chemically defined medium) was prepared according to the method of Letort and Juillard (2001).

### 2.2 Batch fermentations

The cells were harvested by centrifugation (10,000 *g*, 5 min, 4°C) from eighth subcultures. The pellet was washed twice with PBS buffer (50 mmol/L, pH 6.5) and the culture was inoculated in a 10‐l Biotech‐7000 bioreactor (Shanghai Baoxing, Shanghai, China) containing 7 L of CDM. The temperature and rotation speed were fixed at 42.5°C and 150 rpm, respectively. The pH was maintained at 6.25 by the automatic addition of 1 mol/L NaOH. The culture supernatant and cells were obtained by centrifugation (10,000 *g*, 5 min, 4°C) and were preserved at −80°C for further analysis. Batch fermentations were repeated five times. Cell extract preparations were performed according to the methods of Zhengwen et al. (2017).

### 2.3 Analysis methods

The growth rate (μ) of ST‐MZ‐2 was measured spectrophotometrically at 650 nm. The dry weight (DW) of cultured cells was determined gravimetrically. The intracellular pH was measured according to the method of Breeuwer, Drocourt, Rombouts, and Abee (1996). The culture medium was collected and quenched by an equal volume of methanol at −40°C and a reagent containing 50% perchloric acid was used to extract ATP. HPLC with a UV detector and a Sepax HP‐C18 column (250 × 4.6 mm, 5 μm) was used to analyze the intracellular ATP concentration (Ying et al., 2015). The H^+^‐ATPase activity was measured using a commercial kit (Genmed Scientifics Inc., Wilmington, DE, USA). The protein content of the cell‐free extracts was determined with a protein assay kit (Bio‐Rad, Munich, Germany). NADH and NAD^+^ were quantified using HPLC with a ZORBAX SB‐Aq column (250 × 4.6 mm, Agilent) and a UV detector (Ningzi et al., 2013).

To measure whole‐cell amino acids composition, the biomass was hydrolyzed with 6 mol/L of HCl for 20 hr at 120°C. Amino acids were measured by an amino acid analyzer (Acquity UPLC; Waters Corp., Milford, MA). Lactose was quantified by HPLC using a chromatographic system composed of an Alliance 2695 module injector (Waters, Milford, MA, USA), a 2414 Differential Refractometer detector (Waters), and an ICSep ION‐300 ion exchange column (300 × 7.8 mm, 7 μm), all controlled with Empower software (Waters) described by Del et al. (2015). The folic acid, riboflavin, calcium pantothenate, ascorbic acid, biotin, vitamin B_6_, thiamin‐HCl, and cyanocobalamine concentrations were determined by RP‐HPLC with ultraviolet detector (Heudi, Kilinc, & Fontannaz, 2005). *p*‐Amino benzoic acid and nicotinic acid concentrations were determined by HPLC and RP‐HPLC, respectively (Ciulu et al., 2011; Okdeh, Mostafa, & Traboulssie, 2003). The adenine, guanine, xanthine, inosine, and uracil concentrations were measured by LC–MS/MS (Charlotte et al., 2014; Raquel, Sergio, José, & Gilberto, 2007). The concentration of ions was determined using inductively coupled plasma–atomic emission spectrometry (Tu, Zhao, Xu, Li, & Du, 2013).

## 3 RESULTS

### 3.1 Changes in intracellular pH, ATP concentration, H^+^‐ATPase activity, NADH/NAD^+^, and NH_3_ concentration

**Figure 1:**
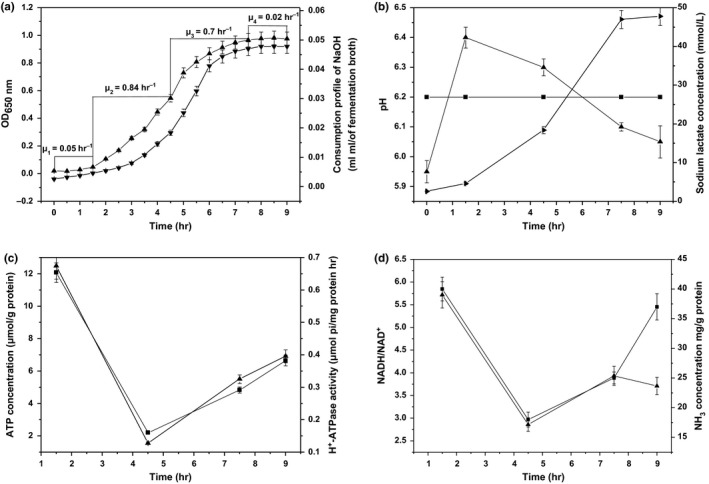
(a) Growth curve (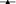) and NaOH consumption profile (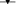) of ST‐MZ‐2 in CDM. (b) Changes in intracellular pH (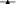), extracellular pH (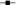), and sodium lactate concentration (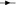) with culture time. (c) Changes in ATP concentration (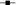) and H^+^‐ATPase activity (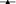) with culture time. (d) Changes in NADH/NAD ^+^ (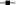) and NH_3_ concentration (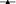) with culture time

In this study, sampling points were defined and the growth phase of ST‐MZ‐2 was divided into four stages (Figure 1a), including lag phase (T1), end‐lag phase to midexponential growth phase (T2), midexponential to end‐exponential growth phase (T3), and end‐exponential growth phase to stationary phase (T4). The intracellular pH of ST‐MZ‐2 increased from 5.95 to 6.41 at lag phase and then decreased from 6.41 to 6.05 during the later fermentation time (Figure 1b). The concentration of sodium lactate increased from 2.61 to 47.67 mmol/L during culture (Figure 1b). The intracellular ATP concentration and H^+^‐ATPase activity were highest at end‐lag phase and gradually increased from midexponential growth phase to stationary phase (Figure 1c). The NADH/NAD^+^ ratios were highest at end‐lag phase, lowest at midexponential growth phase and increased from midexponential growth phase to end‐exponential growth phase before decreasing from end‐exponential growth phase to stationary phase. The intracellular NH_3_ concentration decreased from end‐lag phase to midexponential growth phase and then increased from midexponential growth phase to stationary phase (Figure 1d).

### 3.2 Amino acids

**Figure 2:**
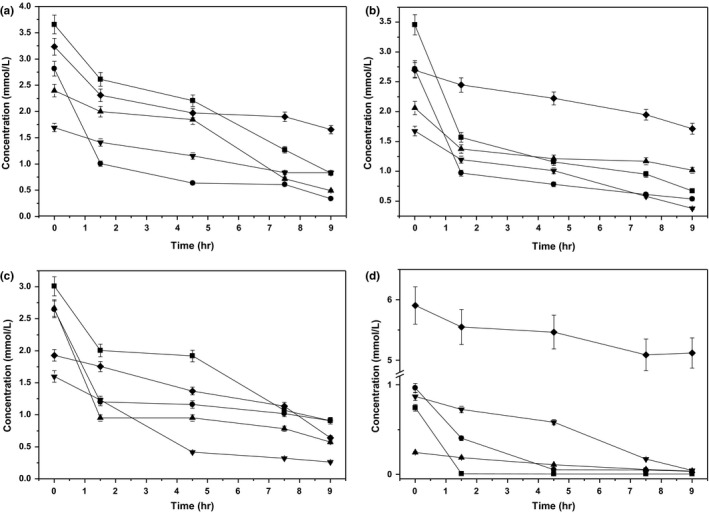
Changes in amino acids concentrations in ST‐MZ‐2 with culture time. (a) Leu (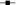), Val (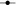), Gly (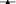), Phe (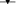), and Ser (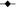). (b) Asp (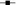), Glu (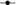), Cys (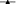), Ile (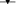), and Ala (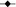). (c) Lys (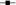), Aan (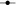), Gln (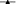), Tyr (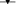), and Thr (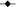). (d) Arg (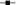), His (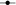), Trp (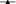), Met (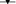), and Pro (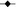)

**Figure 3:**
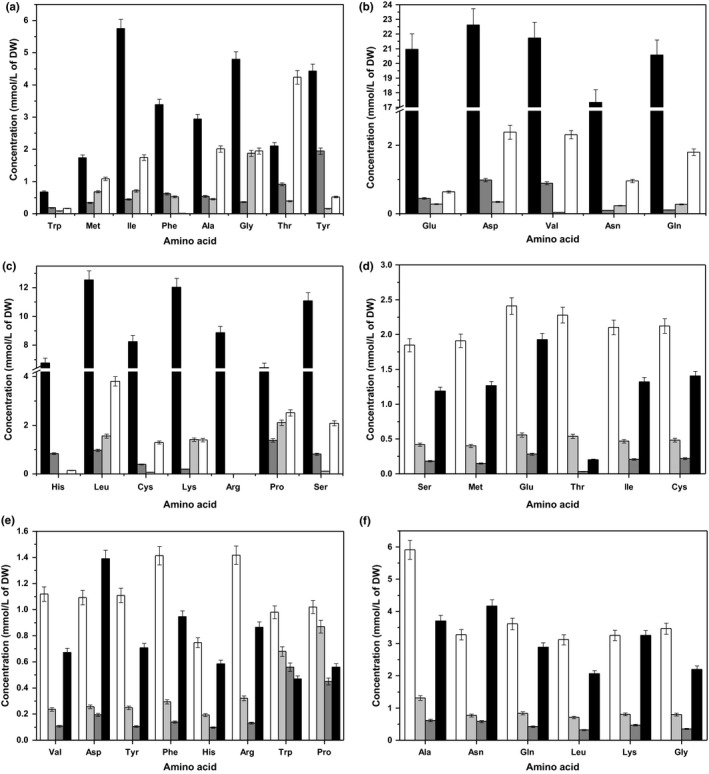
(a, b, and c) Amino acids consumption patterns of ST‐MZ‐2 at different growth stages (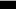
T1; 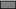
T2; 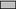
T3; 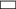
T4). (d, e, and f) Amino acids necessary patterns of ST‐MZ‐2 at different growth stages (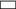
T1; 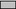
T2; 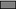
T3; 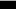
T4)

Figure 2a–d shows the changes in amino acid concentrations with respect to culture time. The consumption amounts of Leu (2.83 mmol/L) and Asp (2.78 mmol/L) were the greatest, followed by Val (2.48 mmol/L). Trp (0.21 mmol/L) exhibited the lowest consumption. The consumption of other amino acids ranged from 0.74 to 2.18 mmol/L. The consumption amounts of Arg, His, and Met were 99.23%, 96.53%, and 94.81% of the addition amounts, which were greatest, followed by Val (87.92%) and Trp (84.68%). Pro (13.26%) exhibited the lowest consumption ratio. The consumption amounts of other amino acids ranged from 36.33% to 83.55% of the addition amounts. The consumption of most amino acids was greatest at lag phase. The consumption of Met, Pro, Phe, Ala, and Gly was highest at T3. Figure 3a–c shows the changes in amino acids concentrations with respect to biomass at different growth stages. The consumption of Met, Ile, Gly, Asn, Gln, Leu, Cys, Lys, Pro, and Ser decreased as the μ increased from lag phase to midexponential growth phase, indicating that the efficiency of their use increased with increasing μ, as the biomass yield based on consumption increased. The consumption of Ala, Glu, Asp, Val, His, and Tyr exhibited similar trends as the ten aforementioned amino acids from lag phase to midexponential growth phase. In addition, the consumption of these amino acids decreased as the μ decreased from midexponential growth phase to end‐exponential growth phase, showing that their efficient use increased with decreasing μ, as the biomass yield based on consumption decreased. The consumption of these amino acids increased as the μ decreased from end‐exponential growth phase to stationary phase. It was noteworthy that almost all amino acids exhibited high consumption and low efficient use based on the biomass yields at lag phase and stationary phase, since biomass did not increase significantly.

Based on amino acid concentrations in the whole‐cell hydrolysates, requirement patterns of amino acids were investigated with respect to biomass at different growth stages (Figure 3d–f). With respect to the total amino acids, the amount of Ala required was significantly high (12.43%), followed by Asn, Lys, Gln, Gly, Leu, and Glu, which ranged from 5.56% to 9.48% of total amino acids needed, whereas other amino acids had lower requirements. The consumption of Val, Asp, His, Glu, Pro, Ser, Tyr, Arg, and Leu notably exceeded the required amounts, especially that the consumption of Val and Asp, the consumption of which were more than eightfold higher compared with the required amounts. Furthermore, the consumption of Gly, Met, Lys, and Phe was slightly higher than the required amounts. Additionally, Trp and Ala had higher required amounts than was observed to be consumed. The overall consumption of amino acids was approximately threefold higher compared to the amounts required, indicating that there was an excessive consumption of amino acids.

### 3.3 Lactose

**Figure 4:**
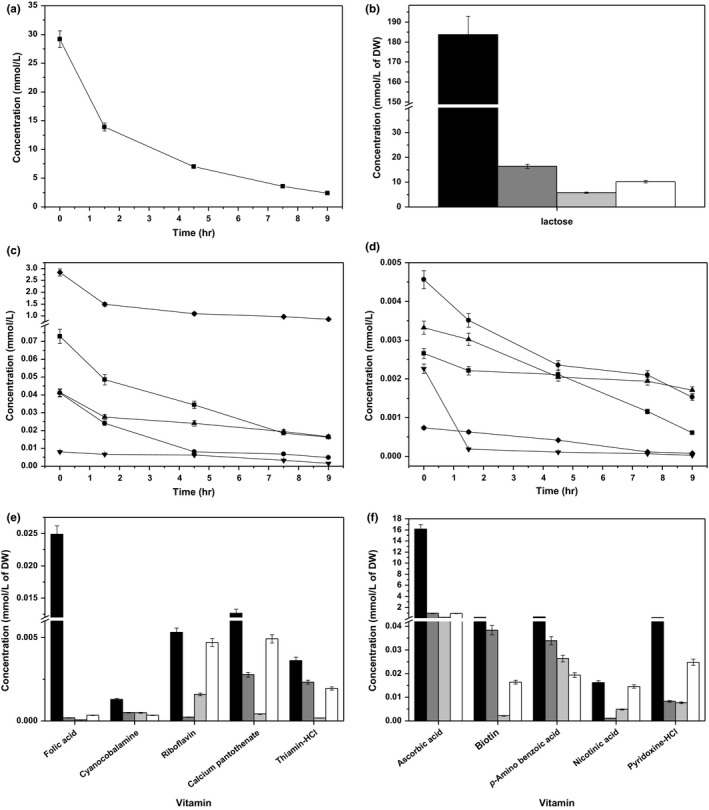
(a) Changes in lactose concentration (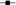) with culture time. (b) Lactose consumption pattern of ST‐MZ‐2 at different growth stages (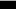
T1; 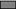
T2; 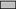
T3; 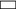
T4). (c) Changes in *p*‐Amino benzoic acid (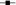), biotin (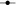), pyridoxine‐HCl (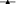), nicotinic acid (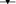), and ascorbic acid (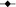) concentrations with culture time. (d) Changes in riboflavin (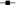), calcium pantothenate (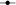), thiamin‐HCl (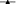), folic acid (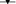), and cyanocobalamine (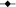) concentrations with culture time. (e and f) Vitamin consumption patterns of ST‐MZ‐2 at different growth stages (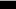
T1; 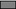
T2; 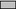
T3; 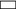
T4)

In Figure 4a and b, the changes in the lactose concentration with respect to the culture time and biomass at different growth stages are shown. The consumption amount of lactose was 26.81 mmol/L and was 91.78% of the addition amount. The highest consumption of lactose occurred at lag phase, and then decreased as the μ increased from end‐lag phase to midexponential growth phase, indicating that the efficiency of its use increased with increasing μ, as biomass yield based on consumption increased. The consumption of lactose decreased as the μ decreased from midexponential growth phase to end‐exponential growth phase, showing that the efficiency of its use increased with decreasing μ, as biomass yield based on consumption decreased. Its consumption increased as μ decreased from end‐exponential growth phase to stationary phase.

### 3.4 Vitamins

In Figure 4c and d, the changes in the concentrations of vitamins are shown with respect to culture time. The most abundantly consumed vitamin was ascorbic acid, with a consumption amount of 1.9727 mmol/L, followed by *p*‐amino benzoic acid, which had consumption amount of 56.6 nmol/L. Cyanocobalamine had the lowest consumption amount (0.7 nmol/L). The consumption amounts of other vitamins ranged from 1.6 to 36.1 nmol/L. The consumption amount of folic acid was 98.68% of the addition amount, which was highest, and thiamin‐HCl (48.49%) was the lowest. The consumption amounts of other vitamins ranged from 59.86% to 89.16% of the addition amounts. Folic acid, ascorbic acid, vitamin B_6_, biotin, and *p*‐amino benzoic acid exhibited the highest consumption at lag phase, whereas calcium pantothenate and thiamine exhibited the highest consumption at T2. Nicotinic acid and riboflavin had the highest consumption at T3. In Figure 4e and f, the changes in the concentrations of vitamins with respect to biomass are shown at different growth stages. The consumption of riboflavin and nicotinic acid decreased as the μ decreased from midexponential growth phase to stationary phase, indicating that they were more effectively utilized at higher μ values, as biomass yield based on consumption increased, with the highest observed efficiency of their use occurring at T2. Additionally, the efficient use of folic acid, calcium pantothenate, thiamine‐HCl, ascorbic acid, biotin and pyridoxine‐HCl was highest at T3, and that of *p*‐amino benzoic acid was highest at stationary phase.

### 3.5 Purines, pyrimidine and inosine

**Figure 5:**
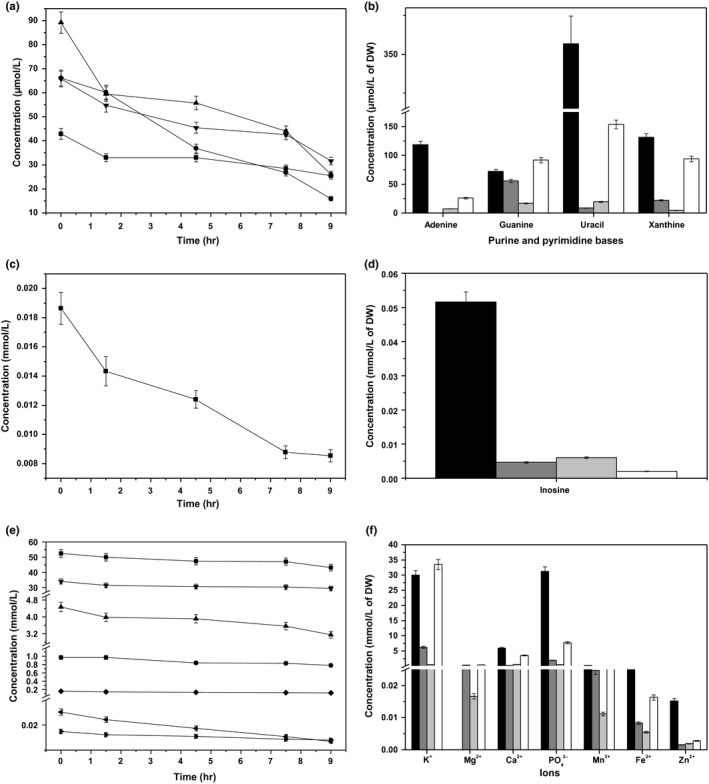
(a) Changes in adenine (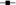), guanine (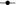), uracil (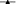), and xanthine (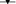) concentrations with culture time. (b) Purine and pyrimidine consumption patterns of ST‐MZ‐2 at different growth stages (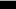
T1; 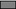 T2; 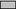 T3; 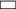 T4). (c) Changes in inosine (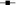 ) concentration with culture time. (d) Inosine consumption pattern of ST‐MZ‐2 at different growth stages (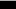
T1; 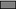
T2; 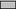
T3; 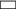
T4). (e) Changes in K^+^ (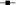), Mg^2+^ (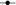), Ca^2+^ (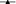), PO_4_^3−^ (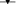), Mn^2+^ (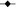), Fe^2+^ (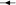), and Zn^2+^ (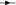) concentrations with respect to culture time. (f) Ions consumption patterns of ST‐MZ‐2 at different growth stages (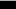
T1; 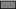
T2; 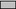
T3; 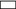
T4)

In Figure 5a, the changes in the concentrations of purines and pyrimidine with respect to culture time are shown. The consumption amount of uracil was the largest (63.20 mmol/L) and was 70.84% of the addition amount. The amount of adenine consumed was lowest (17.40 mmol/L), was 40.56% of the addition amount. Furthermore, the amounts of guanine and xanthine consumed were 50.18 and 34.10 mmol/L, and were 75.83% and 51.87% of the addition amounts, respectively. Adenine and uracil had the highest consumption at lag phase. The phases with the highest consumption of guanine and xanthine were T2 and stationary phase, respectively. In Figure 5b, the changes in the concentrations of purines and pyrimidine with respect to biomass at different growth stages are shown. The biomass utilization efficiency of adenine and uracil was the highest at T2 and lowest at lag phase. Changes in the concentrations of guanine and xanthine with respect to biomass were similar from midexponential growth phase to stationary phase, which increased with decreasing μ, indicating that they were more effectively utilized at higher μ, as biomass yield based on their consumption increased. In Figure 5c and d, the changes in inosine concentration with respect to the culture time and biomass at different growth stages are shown, respectively. The amount and ratio of inosine consumed were 0.01 mmol/L and 54.18%, respectively. The consumption of inosine decreased as μ increased from lag phase to midexponential growth phase. The results indicated that the efficiency of its use increased with increasing μ, as biomass yield based on consumption also increased. Its efficient use was the highest at T2.

### 3.6 Ions

In Figure 5e, the changes in the concentrations of ions are shown with respect to culture time. The most abundantly consumed ion was K^+^ (9.32 mmol/L), which was 17.71% of the addition amounts. The amount of PO_4_^3−^ consumed was 4.6 mmol/L, and was 13.49% of the addition amount, which was the lowest. The consumption amount of Mg^2+^ was 0.19 mmol/L and was 19.59% of the addition amount. The amount of Zn^2+^ consumed (3.4 nmol/L) was the lowest. Fe^2+^ exhibited a low consumption amount, whereas it was 46.44% of the addition amount, which was the highest. The amount of Mn^2+^ and Ca^2+^ consumption was 0.04 and 1.32 mmol/L, and was 24.66% and 29.53% of the addition amounts, respectively. Mn^2+^, Zn^2+^, Ca^2+^ and PO_4_^3−^ exhibited the highest consumption at lag phase, whereas Fe^2+^ and Mg^2+^ exhibited the highest consumption at T2. K^+^ had the highest consumption at stationary phase. In Figure 5f, the changes in the concentrations of ions with respect to biomass at different growth stages are shown. Mg^2+^ and K^+^ showed similar consumption patterns from end‐lag phase to stationary phase. The consumption of PO_4_^3−^, Mn^2+^, and Fe^2+^ decreased with increasing μ, indicating a more effective utilization of these ions at a higher μ with the increasing of biomass as the biomass yield based on consumption increased. In addition, Zn^2+^ and Ca^2+^ had similar consumption patterns from end‐lag phase to stationary phase. Their biomass utilization efficiencies were the highest at T2, whereas that of the other ions was highest at T3. All assayed ions, with the exception of Mg^2+^, exhibited the lowest biomass utilization efficiencies at lag phase and stationary phase.

## 4 DISCUSSION

The intracellular pH of LAB is low when they are initially inoculated into new media but needs to be adjusted to a high intracellular pH during lag phase to grow better during exponential growth phase (Rault, Bouix, & Béal, 2009). Similar results were obtained in this work. One of most expensive ATPase reactions is the translocation of protons across the cytoplasmic membrane, which is catalyzed by H^+^‐ATPase to maintain the intracellular pH (Kilstrup, Hammer, Ruhdal, & Martinussen, 2005). NAD^+^ participates in glycolysis as the coenzyme of dehydrogenase, which brings a large amount of ATP to pump H^+^ out of the cells (Ningzi et al., 2013). The intracellular ATP concentration, H^+^‐ATPase activity, NADH/NAD^+^ ratio, and NH_3_ concentration are highest during lag phase, contributing to the higher intracellular pH during lag phase. The intracellular pH dropped as the sodium lactate concentration increases. This could be attributed to the pH homeostasis by H^+^/lactate symport, which was less effective at high external lactate concentrations (Konings & Otto, 1983). Additionally, the biosynthesis of NH_3_, ATP, H^+^‐ATPase, NAD^+^, and NADH required the consumption of nutrients (Fernández & Zúñiga, 2006; van de Guchte et al., 2002). In this study, almost all nutrients were most efficiently utilized at T2 and T3, showing that these nutrients were primarily used to synthesize biomass. Conversely, the consumption of most nutrients was highest at lag phase, whereas the efficiency of their utilization was lowest, as the biomass increased indistinctively. *S. thermophilus* encounters two stress factors simultaneously during culturing, including the gradual increase in intracellular acidity and extracellular osmotic pressure (Shan et al., 2016; Zhengwen et al., 2017), which inhibit the growth and metabolic activities of LAB by affecting the uptake of nutrients, causing damage to membrane integrity and the synthesis of proteins and nucleic acids (Béal, Marin, Fontaine, Fonseca, & Obert, 2008; Konings & Otto, 1983; Savoie, Champagne, Chiasson, & Audet, 2007). Furthermore, we inferred that changes in stress might result in the efficient utilization of nutrients at T2 and T3, which was lower at lag phase and stationary phase. The excessive consumption of nutrients might be used to counteract stress.

LAB have multiple amino acid requirements to synthesize proteins, provide precursors for the biosynthesis of amino acids, nucleotides, and vitamins, generate metabolic energy, control intracellular pH, and resist stresses (Iyer, Tomar, Maheswari, & Singh, 2010; Savijoki, Ingmer, & Varmanen, 2006). Ala, Asp, Arg, and Glu were observed to be involved in protecting LAB against damage associated with a low pH environment (Ningzi et al., 2013; Senouci‐Rezkallah, Schmitt, & Jobin, 2011). In this study, Arg, Glu, and Asp had the highest consumption at lag phase, whereas Ala had the highest consumption at T3. Furthermore, they were consumed at higher amounts than was requirement. These four amino acids may be involved in the regulation of intracellular acidity at lag phase and T3. Some LAB can metabolize Arg to produce ammonia and ATP to cope with acid stress through pH adjustment and gain an energetic advantage (Vrancken, Rimaux, Wouters, Leroy, & De, 2009). In this study, Arg may have contributed the most to the improvement of intracellular acidity because it was almost exhausted at lag phase. Compared with requirements of amino acids, the extra consumption of amino acids might have been used to resist intracellular and extracellular stresses. *S. thermophilus* was observed to consume a large amount of Leu, Glu, Lys, Ala, Arg, and Asp in M17 medium (Guimont, 2002), which was similarly observed in this study. In addition, the amino acid requirements of LAB are strain dependent, with wide range of differences among species and strains (Letort & Juillard, 2001). Compared with the previous study, similar results were obtained in this study (Hong et al., 2015).

Lactose provided energy and a carbon source for growth of *S. thermophilus* and exhibited the highest consumption at lag phase. The primary role of lactose may be to provide energy for the regulation of intracellular physiological states at lag phase. Vitamins are essential micronutrients that are typically used as precursors of various enzymes that are necessary for vital biochemical reactions in all living cells (LeBlanc et al., 2013). In this study, the mean consumption ratio of vitamins was 75%. The results indicated that ST‐MZ‐2 had a high requirement of vitamins. Ascorbic acid was observed to be an essential growth factor for *Lactobacillus sakei* and *L*. *plantarum*. In addition, biotin was observed to be a stimulatory factor for the growth of some LAB strains or an essential factor for other strains (Hayek & Ibrahim, 2013). In this study, ascorbic acid was consumed in the highest amounts, followed by *p*‐amino benzoic acid, and the consumption rate of folic acid was the highest. Riboflavin, nicotinic acid, and calcium pantothenate were required by *S. thermophilus* and they promote the growth of bacteria (Letort & Juillard, 2001). In this study, the amounts of riboflavin and calcium pantothenate consumed were both high, whereas that of nicotinic acid was low. In addition, some vitamins can replace each other, and individual strains of LAB require one to four vitamins for normal growth (Hayek & Ibrahim, 2013).

Nucleotides are substrates for RNA and DNA synthesis, constituents of coenzymes, are primary energy donors, and activate precursors in peptidoglycan and lipid synthesis (Kilstrup et al., 2005). Some LAB require either purines or pyrimidines. The addition of purines stimulated the growth of *L*. *lactis* MG1363 in chemically defined medium (Martinussen, Wadskov‐Hansen, & Hammer, 2003). In this study, the mean consumption ratio of adenine, guanine, uracil, and xanthine was 60%, and the consumption ratio of inosine was 54.18%. The results showed that ST‐MZ‐2 had a high requirement for purines, pyrimidine, and inosine. It has been reported that the addition of exogenous purine enhanced resistance toward acid stress in *L*. *lactis* (Ryssel et al., 2014). In this study, the consumption of adenine, uracil, and xanthine was highest at lag phase and stationary phase and may be involved in resistance toward acid stress.

Mg^2+^ and Ca^2+^is known to stimulate the growth and improve the survival of *S. thermophilus* (Letort & Juillard, 2001). The acid phosphatase activity can be enhanced by both Ca^2+^ and Mg^2+^, more so by Ca^2+^ (Tham et al., 2010). In this study, the consumption of Mg^2+^ and Ca^2+^ was highest at T2 and T3, respectively. Their consumption exhibited a complementary trend at these two stages. Furthermore, ST‐MZ‐2 required Mg^2+^ and Ca^2+^ to stimulate its growth at T2 and T3. Mn^2+^ has been observed to enhance β‐glucosidase activity (Jeng et al., 2011). In this study, Mn^2+^ had the highest biomass utilization efficiency. The acid phosphatase and β‐glucosidase activity was inhibited by Fe^2+^ and Zn^2+^ (Jeng et al., 2011; Tham et al., 2010). The removal of Fe^2+^ and Zn^2+^ from CDM did not affect the growth of *S. thermophilus* (Letort & Juillard, 2001). Because of the negative effects of these ions, and the low amount and ratio of their consumption observed in this study, they should be reduced or removed from media. The presence of inorganic phosphate in the form of dipotassium phosphate in MRS was observed to markedly reduce the activities of phosphatases (Palacios, Haros, Rosell, & Sanz, 2005). Despite the high consumption, residual concentrations of phosphate were observed to be high in this study. Thus, the phosphate concentration should be reduced or replaced by organic phosphorus.

The ratios of nutrients are crucial for high cell density cultivation of LAB. Shortage of nutrients limits the growth of LAB (Lahtvee et al., 2011). Therefore, it is of crucial importance to include balanced amounts of nutrients in LAB culture media to ensure a suitable level of growth. In this study, nutrients with high consumption ratios and low residual concentrations, such as Arg, Met, His, Trp, Val, lactose, cyanocobalamine, folic acid, biotin, guanine and uracil, were observed during the growth of ST‐MZ‐2. Their concentrations may be unfit for the growth of ST‐MZ‐2 and should be increased in the media. The excessively high initial concentration of nutrients may inhibit growth of LAB (Shan et al., 2016). Nutrients with low consumption ratios and high residual concentrations, such as Pro, Ala, Ser, and most ions, were observed during the growth of ST‐MZ‐2. They may have been present in excess and should be decreased in the media. Additionally, the addition of nutrients with special physiological roles should be considered for the media.

The optimization of balanced amounts of nutrients is important in the design of ideal medium for the high cell density cultivation of LAB. More importantly, we believed that biosynthesis of inadequate or unnecessary nutrients could be avoided in LAB by optimization of balanced amounts of nutrients. Studies have shown that some amino acids, vitamins, and nucleic acid bases are unnecessary for the growth of *S. thermophilus* (Letort & Juillard, 2001). In this study, various amounts of unnecessary nutrients were consumed by ST‐MZ‐2, with some unnecessary nutrients exhibiting high consumption amounts and ratios. Some nonessential nutrients are conducive to the growth of *S. thermophilus* (Hong et al., 2015; Law & Kolstad, 1983). Thus, nonessential nutrients are also required by *S. thermophilus* for better growth. *S. thermophilus* has the capability to synthetize nonessential nutrients using other nutrients (Hayek & Ibrahim, 2013). However, we hypothesized that ideal media for high cell density cultivation should supply various nutrients with more suitable ratios for the growth of LAB, since the synthesis of nutrients requires the consumption of other nutrients, with energy consumption and production of useless intermediate metabolites (Barton, Delneri, Oliver, Rattray, & Bergman, 2010). For example, some organisms can produce vitamins if needed but probably prefer to take up the compounds when available in the environment, because their synthesis typically requires more metabolic energy than transport (Jaehme & Slotboom, 2015). Designing principles for properly balanced media and reducing nutrient waste by controlling ratios of nutritional components in the growth media will be the focus of our future research.

## CONFLICT OF INTEREST

It should be understood that none of the authors have any financial or scientific conflict of interest with regard to the research described in this manuscript.
